# Supplementary material for: Genomic Screening to Identify Food Trees Potentially Dispersed by Precolonial Indigenous Peoples
Source: Genes (Basel). 2022 Mar 8;13(3):476. doi: 10.3390/genes13030476 (PMC8954434; doi:10.3390/genes13030476)
Supplement: Supplementary file 1 [file genes-13-00476-s001.zip › Table_S1.pdf]

Family	Species	Site	Sample (n)	Latitude	Longitude	Region	Voucher	DArTseq Genotyping
**Lauraceae**	*Beilschmiedia bancroftii*	UTR	5	-17.84792	145.5854	SBMC	NSW1031602	Novel
**Lauraceae**	*B. bancroftii*	MtB	2	-17.31579	145.42	SBMC	NSW1008231	Novel
**Lauraceae**	*B. bancroftii*	LR	6	-17.12166	145.6311	SBMC	NSW1008125	Novel
**Lauraceae**	*B. bancroftii*	MtL	6	-16.59479	145.2829	NBMC	NSW1008753	Novel
**Lauraceae**	*B. bancroftii*	MtW	5	-16.25905	145.0607	NBMC	NSW1008354	Novel
**Lauraceae**	*Endiandra globosa*	BH	12	-28.53068	153.5395	NNSW	NSW1025109	Novel
**Lauraceae**	*E. globosa*	HS	12	-28.26058	153.4467	NNSW	NSW1030896	Novel
**Lauraceae**	*E. globosa*	WT	6	-17.60994	145.7661	SBMC	NSW1030906	Novel
**Lauraceae**	*E. globosa*	WC	5	-17.60874	145.7938	SBMC	NSW1035623	Novel
**Lauraceae**	*E. globosa*	WBa	4	-17.52669	145.8292	SBMC	NSW1031627	Novel
**Lauraceae**	*E. globosa*	BKL	8	-17.25306	145.9067	SBMC	NSW1025113	Novel
**Lauraceae**	*E. discolor*	MtS	1	-19.00415	146.2085	SBMC	NSW1030892	Novel
**Lauraceae**	*E. discolor*	BO	3	-19.22741	146.4037	SBMC	NSW1030893	Novel
**Lauraceae**	*E. discolor*	By	10	-22.79032	150.7569	CQLD	NSW1030888	Novel
**Lauraceae**	*E. discolor*	Bu	10	-24.61385	151.5369	SEQ	NSW1035599	Novel
**Lauraceae**	*E. discolor*	C	11	-25.94837	153.0924	SEQ	NSW1035587	Novel
**Lauraceae**	*E. discolor*	HS	5	-28.25497	153.4423	NCRC	NSW1035584	Novel
**Lauraceae**	*E. discolor*	BH	11	-28.53068	153.5395	NCRC	NSW823695	Novel
**Lauraceae**	*E. discolor*	Ni	12	-28.64077	153.3347	NCRC	NSW814436	Novel
**Lauraceae**	*E. discolor*	U	11	-30.24675	153.0901	SCRC	NSW1035573	Novel
**Lauraceae**	*E. compressa*	T	7	-16.1414	145.2536	NBMC	NSW1032692	Novel
**Lauraceae**	*E. compressa*	BKL	6	-17.25306	145.9067	SBMC	NSW1032685	Novel
**Lauraceae**	*E. compressa*	WBa	4	-17.52669	145.8292	SBMC	NSW1031625	Novel
**Lauraceae**	*E. compressa*	WT	10	-17.60994	145.7661	SBMC	NSW1032706	Novel
**Lauraceae**	*E. insignis*	B	5	-17.86508	145.7465	SBMC	NSW1030914	Novel
**Lauraceae**	*E. insignis*	WG	9	-17.6033	145.7688	SBMC	NSW1025112	Novel
**Lauraceae**	*E. insignis*	CF	9	-17.1773	145.66	SBMC	NSW1030925	Novel
**Lauraceae**	*E. insignis*	T	1	-16.1414	145.2536	NBMC	NSW1030931	Novel
**Lauraceae**	*E. pubens*	HH	10	-28.67112	153.5855	NCRC	NSW823686	Novel
**Lauraceae**	*E. pubens*	Ni	10	-28.64077	153.3347	NCRC	NSW814261	Novel
**Lauraceae**	*E. pubens*	HS	8	-28.25315	153.4421	NCRC	NSW1025108	Novel
**Lauraceae**	*E. pubens*	Bu	9	-24.61217	151.5368	SEQ	NSW1030938	Novel
**Sapotaceae**	*Pleioluma queenslandica*	BH	10	-28.530683	153.539517	NNSW	NSW1070512	Novel
**Sapotaceae**	*P. queenslandica*	C	9	-25.948367	153.092383	SEQ	NSW1040095	Novel
**Sapotaceae**	*P. queenslandica*	Bu	9	-24.61385	151.536933	CQLD	NSW1070476	Novel
**Sapotaceae**	*P. queenslandica*	By	10	-22.790317	150.756933	CQLD	NSW1070486	Novel
**Sapotaceae**	*P. queenslandica*	Eu	10	-21.146559	148.489259	CQLD	NSW1070473	Novel

**Sapotaceae**	*Planchonella australis*	D	10	-30.37713	152.7267	SCRC	NSW1037092	Novel
**Sapotaceae**	*P. australis*	HH	9	-28.67112	153.5855	NCRC	NSW814259	Novel
**Sapotaceae**	*P. australis*	Ni	10	-28.64077	153.3347	NCRC	NSW814272	Novel
**Sapotaceae**	*P. australis*	MC	10	-26.77875	152.8811	SEQ	NSW1040080	Novel
**Sapotaceae**	*P. australis*	Bu	4	-24.61217	151.5368	CQLD	NSW1040090	Novel
**Sapotaceae**	*Niemeyera whitei*	BH	9	-28.53068	153.5395	NCRC	NSW823685	Novel
**Sapotaceae**	*N. whitei*	U	12	-30.24675	153.0901	SCRC	NSW1037087	Novel
**Sapotaceae**	*N. whitei*	Ni	10			NCRC	NSW814413	Novel
**Sapotaceae**	*N. prunifera*	Cra	11	-21.18604	148.5519	CQLD	NSW1040030	Novel
**Sapotaceae**	*N. prunifera*	WC	3	-17.61202	145.7941	SBMC	NSW1073094	Novel
**Sapotaceae**	*N. prunifera*	WT	1	-17.6048	145.777	SBMC	NSW1079825	Novel
**Sapotaceae**	*N. prunifera*	WG	5	-17.604	145.7619	SBMC	NSW1040024	Novel
**Sapotaceae**	*N. prunifera*	Rav	10	-17.58423	145.4823	SBMC	NSW1037109	Novel
**Sapotaceae**	*N. prunifera*	MtL	4	-16.5291	145.2832	NBMC	NSW1037113	Novel
**Sapotaceae**	*N. prunifera*	CT	10	-16.07769	145.4717	NBMC	NSW1037089	Novel
**Elaeocarpaceae**	*Elaeocarpus grandis*	CRa	9	-21.188	148.5546	CQLD	NSW1080222	Novel
**Elaeocarpaceae**	*E. grandis*	Eu	11	-21.1504	148.4726	CQLD	NSW1080326	Novel
**Elaeocarpaceae**	*E. grandis*	HV	5	-20.92727	149.0482	CQLD	NSW1080239	Novel
**Elaeocarpaceae**	*E. grandis*	Pa	7	-19.01409	146.1763	SBMC	NSW1080246	Novel
**Elaeocarpaceae**	*E. grandis*	Ca	3	-18.20985	145.8072	SBMC	NSW1080184	Novel
**Elaeocarpaceae**	*E. grandis*	Rav	10	-17.64366	145.5006	SBMC	NSW1080193	Novel
**Elaeocarpaceae**	*E. grandis*	MtF	3	-17.55002	145.55	SBMC	NSW1080313	Novel
**Elaeocarpaceae**	*E. grandis*	WE	4	-17.44281	145.8606	SBMC	NSW1079856	Novel
**Elaeocarpaceae**	*E. grandis*	WBF	5	-17.39983	145.8187	SBMC	NSW1080312	Novel
**Elaeocarpaceae**	*E. grandis*	WT	3	-17.39607	145.7643	SBMC	NSW1079882	Novel
**Elaeocarpaceae**	*E. grandis*	WBB	2	-17.34115	145.8698	SBMC	NSW1079860	Novel
**Elaeocarpaceae**	*E. grandis*	BKL	4	-17.30563	145.9157	SBMC	NSW1079871	Novel
**Elaeocarpaceae**	*E. grandis*	CF	4	-17.28157	145.5697	SBMC	NSW1080319	Novel
**Elaeocarpaceae**	*E. grandis*	MtBa	10	-17.2725	145.4292	SBMC	NSW1080202	Novel
**Elaeocarpaceae**	*E. grandis*	AR	3	-17.26743	145.4529	SBMC	NSW1080316	Novel
**Elaeocarpaceae**	*E. grandis*	LR	2	-17.12166	145.6311	SBMC	NSW1080232	Novel
**Elaeocarpaceae**	*E. grandis*	Ju	9	-16.5667	145.3461	NBMC	NSW1080215	Novel
**Elaeocarpaceae**	*E. grandis*	MtL	6	-16.5314	145.2888	NBMC	NSW1079889	Novel
**Elaeocarpaceae**	*E. grandis*	MG	2	-16.47237	145.3321	NBMC	NSW1079886	Novel
**Elaeocarpaceae**	*E. grandis*	Wh	3	-16.38783	145.3323	NBMC	NSW1079873	Novel
**Elaeocarpaceae**	*E. grandis*	MtW	5	-16.24042	144.9704	NBMC	NSW1079849	Novel
**Elaeocarpaceae**	*E. grandis*	Dai	9	-16.05934	145.4624	NBMC	NSW1079899	Novel
**Elaeocarpaceae**	*E. grandis*	MtSo	4	-16.08185	145.4339	NBMC	NSW1079858	Novel
**Elaeocarpaceae**	*E. johnsonii*	MtSo	7	-16.08185	145.4339	NBMC	NSW1080307	Novel
**Elaeocarpaceae**	*E. johnsonii*	WBF	5	-17.39983	145.8187	SBMC	NSW1080311	Novel
**Elaeocarpaceae**	*E. johnsonii*	WT	8	-17.39607	145.7643	SBMC	NSW1080182	Novel
**Elaeocarpaceae**	*E. bancroftii*	WBB	2	-17.34115	145.8698	SBMC	NSW1079838	Novel
**Elaeocarpaceae**	*E. bancroftii*	BKL	1	-17.30563	145.9157	SBMC	NSW1079835	Novel
**Elaeocarpaceae**	*E. bancroftii*	MTR	6	-16.9901	145.8428	SBMC	NSW1079823	Novel
**Elaeocarpaceae**	*E. bancroftii*	BrC	5	-16.97306	145.5869	SBMC	NSW1079840	Novel
**Elaeocarpaceae**	*E. bancroftii*	JCU	5	-16.81663	145.6891	BMC	NSW1080166	Novel
**Elaeocarpaceae**	*E. bancroftii*	MG	3	-16.47237	145.3321	NBMC	NSW1080176	Novel

**Elaeocarpaceae**	*E. bancroftii*	Wh	8	-16.38783	145.3323	NBMC	NSW1079906	Novel
**Elaeocarpaceae**	*E. bancroftii*	MtW	3	-16.24042	144.9704	NBMC	NSW1079841	Novel
**Elaeocarpaceae**	*E. bancroftii*	MtSo	5	-16.08185	145.4339	NBMC	NSW1079843	Novel
**Elaeocarpaceae**	*E. reticulatus*	Ni1	6	-28.5819	153.3771	NCRC	NSW1014468	[1]
**Elaeocarpaceae**	*E. reticulatus*	Ty	5	-28.60646	153.5703	NCRC	NSW1017020	[1]
**Elaeocarpaceae**	*E. reticulatus*	Ni	6	-28.60812	153.3544	NCRC	NSW1016488	[1]
**Elaeocarpaceae**	*E. reticulatus*	BrH	6	-28.68329	153.6077	NCRC	NSW1016632	[1]
**Elaeocarpaceae**	*E. reticulatus*	G	4	-28.81871	151.959	NCRC	NSW1042498	[1]
**Elaeocarpaceae**	*E. reticulatus*	W	6	-28.94329	153.4684	NCRC	NSW1016567	[1]
**Elaeocarpaceae**	*E. reticulatus*	BB	3	-28.96275	152.0328	NCRC	NSW1017720	[1]
**Elaeocarpaceae**	*E. reticulatus*	Br	5	-29.02809	153.4403	NCRC	NSW1015627	[1]
**Elaeocarpaceae**	*E. reticulatus*	WaC	6	-29.47175	152.3218	SCRC	NSW1016819	[1]
**Elaeocarpaceae**	*E. reticulatus*	WaM	6	-29.51562	152.3606	SCRC	NSW1021236	[1]
**Elaeocarpaceae**	*E. reticulatus*	NB	6	-30.09603	152.7406	SCRC	NSW1014117	[1]
**Elaeocarpaceae**	*E. reticulatus*	CC	4	-30.15512	152.5862	SCRC	NSW1010834	[1]
**Elaeocarpaceae**	*E. reticulatus*	JJ	6	-30.23156	152.8024	SCRC	NSW1014152	[1]
**Elaeocarpaceae**	*E. reticulatus*	Or	4	-30.2515	153.1075	SCRC	NSW1010670	[1]
**Elaeocarpaceae**	*E. reticulatus*	Do	5	-30.35807	152.7689	SCRC	NSW1014260	[1]
**Elaeocarpaceae**	*E. reticulatus*	Gl	6	-30.3814	152.9208	SCRC	NSW1014242	[1]
**Elaeocarpaceae**	*Sloanea australis*	Border Ranges	5	-28.39295	153.0578	NCRC	NSW1015333	[1]
**Elaeocarpaceae**	*S. australis*	Mt Warning	6	-28.398	153.2841	NCRC	NSW1011562	[1]
**Elaeocarpaceae**	*S. australis*	Border Ranges 1	5	-28.40596	153.128	NCRC	NSW1016496	[1]
**Elaeocarpaceae**	*S. australis*	Border Ranges 2	6	-28.40907	153.0242	NCRC	NSW933023	[1]
**Elaeocarpaceae**	*S. australis*	Border Ranges 3	5	-28.43749	153.1434	NCRC	NSW1015257	[1]
**Elaeocarpaceae**	*S. australis*	BH	6	-28.53073	153.5439	NCRC	NSW1017025	[1]
**Elaeocarpaceae**	*S. australis*	Huonbrook	6	-28.53311	153.3423	NCRC	NSW1012588	[1]
**Elaeocarpaceae**	*S. australis*	Nightcap N	5	-28.56228	153.3165	NCRC	NSW1025825	[1]
**Elaeocarpaceae**	*S. australis*	Nightcap S	6	-28.64001	153.3345	NCRC	NSW1011291	[1]
**Elaeocarpaceae**	*S. australis*	HH	5	-28.66619	153.5949	NCRC	NSW1016881	[1]
**Elaeocarpaceae**	*S. australis*	Killen Falls	6	-28.7695	153.5245	NCRC	NSW1015346	[1]
**Elaeocarpaceae**	*S. australis*	Davis Scrub	6	-28.86551	153.4047	NCRC	NSW1017925	[1]
**Elaeocarpaceae**	*S. australis*	Bruxner Park	6	-30.24411	153.0987	SCRC	NSW1010798	[1]
**Elaeocarpaceae**	*S. australis*	Do	5	-30.31792	152.8605	SCRC	NSW839251	[1]
**Elaeocarpaceae**	*S. australis*	Pine Creek	5	-30.40116	153.0037	SCRC	NSW1014219	[1]
**Lauraceae**	*Cryptocaria glaucesens*	Nightcap N	5	-28.5499	153.3373	NCRC	NSW1025194	Novel
**Lauraceae**	*C. glaucesens*	Nightcap	6	-28.59947	153.3773	NCRC	NSW1025255	Novel
**Lauraceae**	*C. glaucesens*	Nightcap S	6	-28.63653	153.3733	NCRC	NSW1017936	Novel
**Lauraceae**	*C. glaucesens*	WaC	6	-29.47434	152.3227	SCRC	NSW1025958	Novel
**Lauraceae**	*C. glaucesens*	Hortons Creek	5	-29.99789	152.6823	SCRC	NSW1026843	Novel
**Lauraceae**	*C. glaucesens*	Mt Hyland	6	-30.161	152.4714	SCRC	NSW1026884	Novel
**Lauraceae**	*C. glaucesens*	Never Never	6	-30.35707	152.7791	SCRC	NSW1026603	Novel
**Lauraceae**	*C. glaucesens*	Bellbrook	6	-30.70633	152.5453	SCRC	NSW1026628	Novel
**Lauraceae**	*Neolitsea dealbata*	Border Ranges	5	-28.3926	153.0579	NCRC	NSW1015369	[1]
**Lauraceae**	*N. dealbata*	Mt Warning	6	-28.39792	153.2841	NCRC	NSW1015267	[1]
**Lauraceae**	*N. dealbata*	Border Ranges 1	6	-28.50409	153.1165	NCRC	NSW1017116	[1]
**Lauraceae**	*N. dealbata*	Nightcap N	6	-28.5661	153.3492	NCRC	NSW1016980	[1]
**Lauraceae**	*N. dealbata*	BH	6	-28.70372	153.6123	NCRC	NSW1016999	[1]

**Lauraceae**	*N. dealbata*	Killen Falls	5	-28.76982	153.5244	NCRC	NSW1006547	[1]
**Lauraceae**	*N. dealbata*	NB	5	-30.13842	152.7057	SCRC	NSW1014315	[1]
**Lauraceae**	*N. dealbata*	Bruxner Park	5	-30.24399	153.0981	SCRC	NSW1010901	[1]
**Lauraceae**	*N. dealbata*	Glennifer	6	-30.36645	152.8999	SCRC	NSW1014131	[1]
**Lauraceae**	*N. dealbata*	Junuy Juluum	5	-30.44099	152.6437	SCRC	NSW1010883	[1]
**Lauraceae**	*N. dealbata*	Way Way	6	-30.76761	152.9431	NCRC	NSW1010816	[1]
**Cunoniaceae**	*Ceratopetalum apetalum*	Springbrook	6	-28.21801	153.3011	NCRC	NSW1026285	[1]
**Cunoniaceae**	*C. apetalum*	Nightcap	6	-28.55731	153.3426	NCRC	NSW1026265	[1]
**Cunoniaceae**	*C. apetalum*	Nightcap1	6	-28.56497	153.3364	NCRC	NSW1026268	[1]
**Cunoniaceae**	*C. apetalum*	Washpool N	6	-29.18668	152.4284	CRC	NSW1026288	[1]
**Cunoniaceae**	*C. apetalum*	Washpool C	6	-29.46928	152.3171	SCRC	NSW1025185	[1]
**Cunoniaceae**	*C. apetalum*	NB	6	-30.19831	152.7202	SCRC	NSW1025638	[1]
**Cunoniaceae**	*C. apetalum*	Do	6	-30.35661	152.7894	SCRC	NSW1025684	[1]
**Sapindaceae**	*Diploglottis australis*	Border Ranges	6	-28.3926	153.0579	NCRC	NSW1016872	[1]
**Sapindaceae**	*D. australis*	Border Ranges 1	6	-28.3945	153.1883	NCRC	NSW1015349	[1]
**Sapindaceae**	*D. australis*	Mt Warning	6	-28.40112	153.275	NCRC	NSW1015191	[1]
**Sapindaceae**	*D. australis*	BH	5	-28.53056	153.5444	NCRC	NSW1015217	[1]
**Sapindaceae**	*D. australis*	Nightcap N	5	-28.57027	153.3355	NCRC	NSW1015230	[1]
**Sapindaceae**	*D. australis*	Nightcap S	6	-28.6397	153.3372	NCRC	NSW1017110	[1]
**Sapindaceae**	*D. australis*	HH	6	-28.67106	153.5859	NCRC	NSW1017082	[1]
**Sapindaceae**	*D. australis*	Washpool	5	-29.18662	152.4288	SCRC	NSW838366	[1]
**Sapindaceae**	*D. australis*	Clouds SF	5	-30.13922	152.5969	SCRC	NSW1013656	[1]
**Sapindaceae**	*D. australis*	Orara	6	-30.24427	153.0985	SCRC	NSW1010629	[1]
**Sapindaceae**	*D. australis*	Way Way	5	-30.76767	152.9434	NCRC	NSW1010843	[1]
**Myrtaceae**	*Tristaniopsis laurina*	Border Ranges 3	6	-28.40917	153.0241	NCRC	NSW1015588	[2]
**Myrtaceae**	*T. laurina*	Tweed	6	-28.44103	153.3417	NCRC	NSW1015268	[2]
**Myrtaceae**	*T. laurina*	BarkersVale	6	-28.51724	153.1183	NCRC	NSW1015288	[2]
**Myrtaceae**	*T. laurina*	NightcapLRC	6	-28.64113	153.3366	NCRC	NSW1014617	[2]
**Myrtaceae**	*T. laurina*	KillenFalls	6	-28.76948	153.5234	NCRC	NSW1015558	[2]
**Myrtaceae**	*T. laurina*	Cangai	6	-29.48408	152.447	CRC	NSW1015552	[2]
**Myrtaceae**	*T. laurina*	Nymboida	6	-30.13201	152.7034	SCRC	NSW1014309	[2]
**Myrtaceae**	*T. laurina*	Bucca	6	-30.19144	153.1072	SCRC	NSW1014159	[2]
**Myrtaceae**	*T. laurina*	Bobo	6	-30.22226	152.8299	SCRC	NSW1014304	[2]
**Myrtaceae**	*T. laurina*	Glennifer	6	-30.38705	152.8861	SCRC	NSW1014154	[2]
**Myrtaceae**	*T. laurina*	Bonville	5	-30.39955	153.0059	SCRC	NSW1014169	[2]
**Myrtaceae**	*T. laurina*	Bellinger	6	-30.43847	152.6603	SCRC	NSW1010776	[2]
**Myrtaceae**	*T. laurina*	Bonville	6	-30.59211	153.0063	SCRC	NSW1014167	[2]
**Myrtaceae**	*T. laurina*	WayWay	6	-30.76803	152.9435	NCRC	NSW1010743	[2]
**Myrtaceae**	*T. collina*	Border Ranges 2	6	-28.43729	153.1434	NCRC	NSW1014573	[2]
**Myrtaceae**	*T. collina*	Nightcap	6	-28.56987	153.3436	NCRC	NSW1014662	[2]
**Myrtaceae**	*T. collina*	Moonpar	5	-30.18758	152.6913	SCRC	NSW1014299	[2]
**Myrtaceae**	*T. collina*	Orara West	6	-30.2243	152.9632	SCRC	NSW1010858	[2]
**Myrtaceae**	*T. collina*	Do	5	-30.30735	152.7613	SCRC	NSW1014105	[2]
**Myrtaceae**	*T. collina*	Darkwood	5	-30.44119	152.6433	SCRC	NSW1010761	[2]
**Myrtaceae**	*T. collina*	Jeogla	6	-30.66938	152.2012	SCRC	NSW1010774	[2]
**Lauraceae**	*Beilschmiedia tooram*	B	1	-17.86508	145.7465	SBMC	NSW1031599	Novel
**Lauraceae**	*B. tooram*	UTR	9	-17.69722	145.4977	SBMC	NSW1031605	Novel

**Lauraceae**	*B. tooram*	CF	2	-17.1773	145.66	SBMC	NSW1031588	Novel
**Lauraceae**	*B. tooram*	LR	5	-17.12047	145.6314	SBMC	NSW1031590	Novel
**Lauraceae**	*B. volckii*	WC	8	-17.37954	145.7397	SBMC	NSW1032711	Novel
**Lauraceae**	*B. volckii*	T	10	-16.1414	145.2536	NBMC	NSW1032719	Novel
**Lauraceae**	*Endiandra introrsa*	D	9	-30.37713	152.7267	SCRC	NSW1032673	Novel
**Lauraceae**	*E. introrsa*	Ni	6	-28.5653	153.3371	NCRC	NSW814428	Novel
**Lauraceae**	*E. jonesii*	MtL	5	-16.52965	145.2864	NBMC	NSW1009639	Novel
**Fabaceae**	*Castanospermum australe*	IR	6	-12.7142	143.3209	CYP	NSW1036091	Novel
**Fabaceae**	*C. australe*	Ku	4	-13.8071	143.4704	CYP	NSW1036094	Novel
**Fabaceae**	*C. australe*	CT	7	-16.0693	145.4623	NBMC	NSW1036157	Novel
**Fabaceae**	*C. australe*	ToS	10	-17.2307	145.4799	SBMC	NSW1036113	Novel
**Fabaceae**	*C. australe*	WG	9	-17.6033	145.7688	SBMC	NSW1036175	Novel
**Fabaceae**	*C. australe*	MC	9	-26.7777	152.8806	SEQ	NSW1036059	Novel
**Fabaceae**	*C. australe*	HS	10	-28.2553	153.448	NNSW	NSW1036165	Novel
**Fabaceae**	*C. australe*	Raz	5	-28.4259	153.0018	NNSW	NSW1036031	Novel
**Fabaceae**	*C. australe*	MP	2	-28.4397	152.8813	NNSW	NSW1036038	Novel
**Fabaceae**	*C. australe*	Ni	8	-28.6326	153.3417	NNSW	NSW1035988	Novel
**Fabaceae**	*C. australe*	VP	9	-28.9023	153.4104	NNSW	NSW1036131	Novel
**Fabaceae**	*C. australe*	Ram	2	-29.6521	152.7996	NNSW	NSW1036044	Novel
**Fabaceae**	*C. australe*	OrN	3	-29.7235	152.81	NNSW	NSW1036053	Novel
**Fabaceae**	*C. australe*	Or	4	-29.8258	152.8914	NNSW	NSW1036057	Novel

Table S1. Meta data for the study species, including sample site location and associated herbarium voucher housed at the National Herbarium of NSW (Sydney, Australia).

References

1. Rossetto, M.; Beaumont, L.; Das, S.; Yap, S. Bioclimatic Discordance: Combining Molecular and Environmental Data to Identify Floristic Refugia and Corridors. Final Report Prepared for the NSW Office of Environment and Heritage. April 2018, pp. 1–10. Available online: https://climatechange.environment.nsw.gov.au/
2. Fahey, M.; Rossetto, M.; Wilson, P.D.; Ho, S.Y.W. Habitat preference differentiates the Holocene range dynamics but not barrier effects on two sympatric, congeneric trees (Tristaniopsis, Myrtaceae). *Heredity (Edinb).* **2019**, doi:10.1038/s41437-019-0243-x.
