# Supplementary material for: Genomic Screening to Identify Food Trees Potentially Dispersed by Precolonial Indigenous Peoples
Source: Genes (Basel). 2022 Mar 8;13(3):476. doi: 10.3390/genes13030476 (PMC8954434; doi:10.3390/genes13030476)
Supplement: Supplementary file 1 [file genes-13-00476-s001.zip › Table_S2.pdf]

Table S2. Pairwise genetic and geographic distance values used to calculate each species' Mantel score and construct violin plots of average pairwise Fst estimates amongst species grouped by fruit traits.

Fruit trait (mm)	Family	Species	Site1	Site2	distance (km)	Fst
edible >30	Elaeocarpaceae	*Elaeocarpus bancroftii*	Malbon Thompson Range	Mt Sorrow	110	0.118
edible >30	Elaeocarpaceae	*E. johnsonii*	WooroonooranBF	Mt Sorrow	151	0.220
edible >30	Elaeocarpaceae	*E. johnsonii*	Mt Sorrow	WooroonooranW	150	0.230
edible >30	Elaeocarpaceae	*E. johnsonii*	WooroonooranBF	WooroonooranW	6	0.072
edible <30	Elaeocarpaceae	*E. reticulatus*	Tyagarah	Dorrigo D	209	0.253
edible <30	Elaeocarpaceae	*E. reticulatus*	Nightcap1	Dorrigo D	206	0.150
edible <30	Elaeocarpaceae	*E. reticulatus*	Nightcap	Dorrigo D	203	0.156
edible <30	Elaeocarpaceae	*E. reticulatus*	Broken Head	Dorrigo D	201	0.236
edible <30	Elaeocarpaceae	*E. reticulatus*	Tyagarah	Junuy Jurum	196	0.265
edible <30	Elaeocarpaceae	*E. reticulatus*	Nightcap1	Junuy Jurum	192	0.162
edible <30	Elaeocarpaceae	*E. reticulatus*	Nightcap	Junuy Jurum	188	0.168
edible <30	Elaeocarpaceae	*E. reticulatus*	Broken Head	Junuy Jurum	188	0.249
edible <30	Elaeocarpaceae	*E. reticulatus*	Tyagarah	Nymboi-Binderay	184	0.251
edible <30	Elaeocarpaceae	*E. reticulatus*	Nightcap1	Nymboi-Binderay	179	0.147
edible <30	Elaeocarpaceae	*E. reticulatus*	Broken Head	Nymboi-Binderay	177	0.234
edible <30	Elaeocarpaceae	*E. reticulatus*	Nightcap	Nymboi-Binderay	176	0.154
edible <30	Elaeocarpaceae	*E. reticulatus*	Wardell	Dorrigo D	171	0.266
edible <30	Elaeocarpaceae	*E. reticulatus*	Broadwater	Dorrigo D	161	0.224
edible <30	Elaeocarpaceae	*E. reticulatus*	Wardell	Junuy Jurum	157	0.276
edible <30	Elaeocarpaceae	*E. reticulatus*	Tyagarah	Washpool M	155	0.235
edible <30	Elaeocarpaceae	*E. reticulatus*	Tyagarah	Washpool C	155	0.278
edible <30	Elaeocarpaceae	*E. reticulatus*	Broken Head	Washpool C	152	0.264
edible <30	Elaeocarpaceae	*E. reticulatus*	Broken Head	Washpool M	151	0.224
edible <30	Elaeocarpaceae	*E. reticulatus*	Broadwater	Junuy Jurum	147	0.236
edible <30	Elaeocarpaceae	*E. reticulatus*	Wardell	Nymboi-Binderay	146	0.262
edible <30	Elaeocarpaceae	*E. reticulatus*	Nightcap1	Washpool M	144	0.146
edible <30	Elaeocarpaceae	*E. reticulatus*	Nightcap1	Washpool C	143	0.179
edible <30	Elaeocarpaceae	*E. reticulatus*	Nightcap	Washpool M	140	0.150
edible <30	Elaeocarpaceae	*E. reticulatus*	Nightcap	Washpool C	139	0.185
edible <30	Elaeocarpaceae	*E. reticulatus*	Broadwater	Nymboi-Binderay	137	0.223
edible <30	Elaeocarpaceae	*E. reticulatus*	Wardell	Washpool C	126	0.291
edible <30	Elaeocarpaceae	*E. reticulatus*	Wardell	Washpool M	125	0.245
edible <30	Elaeocarpaceae	*E. reticulatus*	Broadwater	Washpool C	119	0.251

edible <30	Elaeocarpaceae	*E. reticulatus*	Broadwater	Washpool M	118	0.211
edible <30	Elaeocarpaceae	*E. reticulatus*	Washpool C	Dorrigo D	109	0.104
edible <30	Elaeocarpaceae	*E. reticulatus*	Washpool M	Dorrigo D	103	0.078
edible <30	Elaeocarpaceae	*E. reticulatus*	Washpool C	Junuy Jurum	96	0.113
edible <30	Elaeocarpaceae	*E. reticulatus*	Washpool M	Junuy Jurum	90	0.088
edible <30	Elaeocarpaceae	*E. reticulatus*	Washpool C	Nymboi-Binderay	80	0.107
edible <30	Elaeocarpaceae	*E. reticulatus*	Washpool M	Nymboi-Binderay	74	0.088
edible <30	Elaeocarpaceae	*E. reticulatus*	Nightcap1	Broadwater	50	0.211
edible <30	Elaeocarpaceae	*E. reticulatus*	Tyagarah	Broadwater	49	0.129
edible <30	Elaeocarpaceae	*E. reticulatus*	Nightcap	Broadwater	48	0.221
edible <30	Elaeocarpaceae	*E. reticulatus*	Nightcap1	Wardell	41	0.245
edible <30	Elaeocarpaceae	*E. reticulatus*	Broken Head	Broadwater	40	0.120
edible <30	Elaeocarpaceae	*E. reticulatus*	Nightcap	Wardell	39	0.255
edible <30	Elaeocarpaceae	*E. reticulatus*	Tyagarah	Wardell	39	0.153
edible <30	Elaeocarpaceae	*E. reticulatus*	Broken Head	Wardell	30	0.137
edible <30	Elaeocarpaceae	*E. reticulatus*	Nymboi-Binderay	Dorrigo D	30	0.039
edible <30	Elaeocarpaceae	*E. reticulatus*	Nightcap	Broken Head	26	0.222
edible <30	Elaeocarpaceae	*E. reticulatus*	Nightcap1	Broken Head	26	0.209
edible <30	Elaeocarpaceae	*E. reticulatus*	Tyagarah	Nightcap	21	0.240
edible <30	Elaeocarpaceae	*E. reticulatus*	Nightcap1	Tyagarah	19	0.224
edible <30	Elaeocarpaceae	*E. reticulatus*	Nymboi-Binderay	Junuy Jurum	16	0.038
edible <30	Elaeocarpaceae	*E. reticulatus*	Junuy Jurum	Dorrigo D	14	0.048
edible <30	Elaeocarpaceae	*E. reticulatus*	Tyagarah	Broken Head	11	0.074
edible <30	Elaeocarpaceae	*E. reticulatus*	Wardell	Broadwater	10	0.124
edible <30	Elaeocarpaceae	*E. reticulatus*	Washpool C	Washpool M	6	0.062
edible <30	Elaeocarpaceae	*E. reticulatus*	Nightcap1	Nightcap	4	0.031
edible >30	Elaeocarpaceae	*E.bancroftii*	Bridle Creek	Mt Sorrow	101	0.114
edible >30	Elaeocarpaceae	*E.bancroftii*	Malbon Thompson Range	Whyanbeel	86	0.114
edible >30	Elaeocarpaceae	*E.bancroftii*	JCU	Mt Sorrow	86	0.099
edible >30	Elaeocarpaceae	*E.bancroftii*	Bridle Creek	Whyanbeel	71	0.073
edible >30	Elaeocarpaceae	*E.bancroftii*	JCU	Whyanbeel	61	0.062
edible >30	Elaeocarpaceae	*E.bancroftii*	Mt Windsor	Mt Sorrow	53	0.100
edible >30	Elaeocarpaceae	*E.bancroftii*	Mossman Gorge	Mt Sorrow	45	0.098
edible >30	Elaeocarpaceae	*E.bancroftii*	Whyanbeel	Mt Sorrow	36	0.073
edible >30	Elaeocarpaceae	*E.bancroftii*	Malbon Thompson Range	Bridle Creek	27	0.147
edible >30	Elaeocarpaceae	*E.bancroftii*	Malbon Thompson Range	JCU	25	0.115

edible >30	Elaeocarpaceae	*E.bancroftii*	Bridle Creek	JCU	21	0.077
edible >30	Elaeocarpaceae	*E.bancroftii*	Mossman Gorge	Whyanbeel	9	0.060
edible >30	Fabaceae	*Castanospermum australe*	Iron Range NP	Gooligan Creek	605	0.233
edible >30	Fabaceae	*C. australe*	Iron Range NP	South Tolga Scrub	544	0.250
edible >30	Fabaceae	*C. australe*	Iron Range NP	Cape Tribulation	440	0.237
edible >30	Fabaceae	*C. australe*	Mary Cairncross	Orara	334	0.139
edible >30	Fabaceae	*C. australe*	Mary Cairncross	Victoria Park III	242	0.210
edible >30	Fabaceae	*C. australe*	Mary Cairncross	Big Scrub	211	0.173
edible >30	Fabaceae	*C. australe*	Mary Cairncross	Moore Park	185	0.117
edible >30	Fabaceae	*C. australe*	Mary Cairncross	Razorback	184	0.123
edible >30	Fabaceae	*C. australe*	Hogans Scrub	Orara	179	0.159
edible >30	Fabaceae	*C. australe*	Cape Tribulation	Gooligan Creek	174	0.193
edible >30	Fabaceae	*C. australe*	Mary Cairncross	Hogans Scrub	174	0.138
edible >30	Fabaceae	*C. australe*	Razorback	Orara	152	0.119
edible >30	Fabaceae	*C. australe*	Moore Park	Orara	149	0.125
edible >30	Fabaceae	*C. australe*	Big Scrub	Orara	136	0.188
edible >30	Fabaceae	*C. australe*	Victoria Park III	Orara	112	0.218
edible >30	Fabaceae	*C. australe*	Cape Tribulation	South Tolga Scrub	110	0.205
edible >30	Fabaceae	*C. australe*	Moore Park	Victoria Park III	73	0.190
edible >30	Fabaceae	*C. australe*	Hogans Scrub	Victoria Park III	72	0.123
edible >30	Fabaceae	*C. australe*	Razorback	Victoria Park III	66	0.180
edible >30	Fabaceae	*C. australe*	South Tolga Scrub	Gooligan Creek	64	0.094
edible >30	Fabaceae	*C. australe*	Hogans Scrub	Moore Park	59	0.119
edible >30	Fabaceae	*C. australe*	Moore Park	Big Scrub	50	0.155
edible >30	Fabaceae	*C. australe*	Hogans Scrub	Razorback	48	0.116
edible >30	Fabaceae	*C. australe*	Hogans Scrub	Big Scrub	43	0.088
edible >30	Fabaceae	*C. australe*	Razorback	Big Scrub	41	0.148
edible >30	Fabaceae	*C. australe*	Big Scrub	Victoria Park III	31	0.118
edible >30	Fabaceae	*C. australe*	Razorback	Moore Park	12	0.045
edible >30	Lauraceae	*Beilschmiedia bancroftii*	Upper Tully Range	Mt Windsor	175	0.191
edible >30	Lauraceae	*B. bancroftii*	Upper Tully Range	Mt Lewis	131	0.047
edible >30	Lauraceae	*B. bancroftii*	Lamb Range	Mt Windsor	114	0.169
edible >30	Lauraceae	*B. bancroftii*	Upper Tully Range	Lamb Range	71	0.062
edible >30	Lauraceae	*B. bancroftii*	Lamb Range	Mt Lewis	68	0.047
edible >30	Lauraceae	*B. bancroftii*	Mt Lewis	Mt Windsor	46	0.159

edible >30	Lauraceae	*B. tooram*	Upper Tully Range	Lamb Range	64	0.056
edible >30	Lauraceae	*B. volckii*	Wooroonooran C	Tulip	147	0.350
edible <30	Lauraceae	*Cryptocaria glaucesens*	Nightcap	Never Never	204	0.179
edible <30	Lauraceae	*C. glaucesens*	Nightcap S	Never Never	200	0.169
edible <30	Lauraceae	*C. glaucesens*	Nightcap N	Mt Hyland	198	0.196
edible <30	Lauraceae	*C. glaucesens*	Nightcap	Mt Hyland	195	0.186
edible <30	Lauraceae	*C. glaucesens*	Nightcap S	Mt Hyland	191	0.177
edible <30	Lauraceae	*C. glaucesens*	Nightcap N	Hortons Creek	173	0.157
edible <30	Lauraceae	*C. glaucesens*	Nightcap	Hortons Creek	170	0.148
edible <30	Lauraceae	*C. glaucesens*	Nightcap S	Hortons Creek	166	0.140
edible <30	Lauraceae	*C. glaucesens*	Nightcap N	Washpool C	143	0.190
edible <30	Lauraceae	*C. glaucesens*	Nightcap	Washpool C	141	0.180
edible <30	Lauraceae	*C. glaucesens*	Nightcap S	Washpool C	138	0.171
edible <30	Lauraceae	*C. glaucesens*	Washpool C	Never Never	107	0.053
edible <30	Lauraceae	*C. glaucesens*	Washpool C	Mt Hyland	78	0.062
edible <30	Lauraceae	*C. glaucesens*	Hortons Creek	Never Never	41	0.093
edible <30	Lauraceae	*C. glaucesens*	Mt Hyland	Never Never	36	0.047
edible <30	Lauraceae	*C. glaucesens*	Hortons Creek	Mt Hyland	27	0.106
edible <30	Lauraceae	*C. glaucesens*	Nightcap N	Nightcap S	10	0.034
edible <30	Lauraceae	*C. glaucesens*	Nightcap N	Nightcap	7	0.039
edible <30	Lauraceae	*C. glaucesens*	Nightcap	Nightcap S	4	0.031
edible <30	Lauraceae	*C. glaucesens*	Nightcap N	Never Never	209	0.187
edible >30	Lauraceae	*Endiandra compressa*	Tulip	Wooroonooran South	172	0.301
edible >30	Lauraceae	*E. compressa*	Tulip	Bellenden Ker Lowlands	142	0.358
edible >30	Lauraceae	*E. compressa*	Bellenden Ker Lowlands	Wooroonooran South	42	0.125
edible <30	Lauraceae	*E. discolor*	Byfield	Ulidarra	862	0.515
edible <30	Lauraceae	*E. discolor*	Byfield	Nightcap	701	0.506
edible <30	Lauraceae	*E. discolor*	Byfield	Brunswick Heads	697	0.523
edible <30	Lauraceae	*E. discolor*	Byfield	Hogan's Scrub	665	0.505
edible <30	Lauraceae	*E. discolor*	Bulburin	Ulidarra	646	0.397
edible <30	Lauraceae	*E. discolor*	Bulburin	Nightcap	483	0.387
edible <30	Lauraceae	*E. discolor*	Bulburin	Brunswick Heads	479	0.408
edible <30	Lauraceae	*E. discolor*	Cooloola	Ulidarra	478	0.088
edible <30	Lauraceae	*E. discolor*	Bulburin	Hogan's Scrub	448	0.377

edible <30	Lauraceae	*E. discolor*	Byfield	Cooloola	424	0.504
edible <30	Lauraceae	*E. discolor*	Cooloola	Nightcap	301	0.079
edible <30	Lauraceae	*E. discolor*	Cooloola	Brunswick Heads	291	0.108
edible <30	Lauraceae	*E. discolor*	Cooloola	Hogan's Scrub	259	0.101
edible <30	Lauraceae	*E. discolor*	Hogan's Scrub	Ulidarra	224	0.069
edible <30	Lauraceae	*E. discolor*	Byfield	Bulburin	218	0.134
edible <30	Lauraceae	*E. discolor*	Bulburin	Cooloola	216	0.385
edible <30	Lauraceae	*E. discolor*	Brunswick Heads	Ulidarra	196	0.061
edible <30	Lauraceae	*E. discolor*	Nightcap	Ulidarra	180	0.031
edible <30	Lauraceae	*E. discolor*	Hogan's Scrub	Nightcap	44	0.057
edible <30	Lauraceae	*E. discolor*	Hogan's Scrub	Brunswick Heads	32	0.091
edible <30	Lauraceae	*E. discolor*	Brunswick Heads	Nightcap	23	0.047
edible >30	Lauraceae	*E. globosa*	Tchupala	Bellenden Ker Lowlands	42	0.232
edible >30	Lauraceae	*E. globosa*	Crawfords	Bellenden Ker Lowlands	41	0.250
edible >30	Lauraceae	*E. globosa*	Barong	Bellenden Ker Lowlands	32	0.254
edible >30	Lauraceae	*E. globosa*	Brunswick Heads	Hogans Scrub	31	0.095
edible >30	Lauraceae	*E. globosa*	Tchupala	Barong	11	0.107
edible >30	Lauraceae	*E. globosa*	Crawfords	Barong	10	0.126
edible >30	Lauraceae	*E. globosa*	Tchupala	Crawfords	3	0.078
edible <30	Lauraceae	*Elaeocarpus grandis*	Hidden Valley	Mt Windsor	676	0.281
edible <30	Lauraceae	*E. grandis*	Clarke Range	Mt Windsor	668	0.217
edible <30	Lauraceae	*E. grandis*	Hidden Valley	Daintree	661	0.258
edible <30	Lauraceae	*E. grandis*	Eungella	Mt Windsor	659	0.201
edible <30	Lauraceae	*E. grandis*	Clarke Range	Daintree	657	0.205
edible <30	Lauraceae	*E. grandis*	Eungella	Daintree	649	0.188
edible <30	Lauraceae	*E. grandis*	Hidden Valley	Mt Lewis	630	0.261
edible <30	Lauraceae	*E. grandis*	Hidden Valley	Julatten	623	0.269
edible <30	Lauraceae	*E. grandis*	Clarke Range	Mt Lewis	622	0.197
edible <30	Lauraceae	*E. grandis*	Clarke Range	Julatten	615	0.211
edible <30	Lauraceae	*E. grandis*	Eungella	Mt Lewis	613	0.183
edible <30	Lauraceae	*E. grandis*	Eungella	Julatten	607	0.198
edible <30	Lauraceae	*E. grandis*	Hidden Valley	Mt Baldy	557	0.251
edible <30	Lauraceae	*E. grandis*	Clarke Range	Mt Baldy	546	0.181
edible <30	Lauraceae	*E. grandis*	Eungella	Mt Baldy	537	0.168
edible <30	Lauraceae	*E. grandis*	Hidden Valley	Ravenshoe S	522	0.211

edible <30	Lauraceae	*E. grandis*	Hidden Valley	Bartle Frere	519	0.235
edible <30	Lauraceae	*E. grandis*	Clarke Range	Bartle Frere	510	0.164
edible <30	Lauraceae	*E. grandis*	Clarke Range	Ravenshoe S	508	0.145
edible <30	Lauraceae	*E. grandis*	Eungella	Bartle Frere	502	0.150
edible <30	Lauraceae	*E. grandis*	Eungella	Ravenshoe S	499	0.135
edible <30	Lauraceae	*E. grandis*	Hidden Valley	Paluma	368	0.224
edible <30	Lauraceae	*E. grandis*	Clarke Range	Paluma	347	0.150
edible <30	Lauraceae	*E. grandis*	Eungella	Paluma	338	0.137
edible <30	Lauraceae	*E. grandis*	Paluma	Daintree	338	0.111
edible <30	Lauraceae	*E. grandis*	Paluma	Mt Windsor	334	0.115
edible <30	Lauraceae	*E. grandis*	Paluma	Mt Lewis	292	0.102
edible <30	Lauraceae	*E. grandis*	Paluma	Julatten	286	0.119
edible <30	Lauraceae	*E. grandis*	Paluma	Mt Baldy	209	0.087
edible <30	Lauraceae	*E. grandis*	Paluma	Bartle Frere	184	0.060
edible <30	Lauraceae	*E. grandis*	Ravenshoe S	Daintree	176	0.084
edible <30	Lauraceae	*E. grandis*	Paluma	Ravenshoe S	168	0.051
edible <30	Lauraceae	*E. grandis*	Ravenshoe S	Mt Windsor	166	0.087
edible <30	Lauraceae	*E. grandis*	Bartle Frere	Mt Windsor	158	0.088
edible <30	Lauraceae	*E. grandis*	Bartle Frere	Daintree	154	0.078
edible <30	Lauraceae	*E. grandis*	Mt Baldy	Daintree	135	0.113
edible <30	Lauraceae	*E. grandis*	Ravenshoe S	Mt Lewis	126	0.074
edible <30	Lauraceae	*E. grandis*	Mt Baldy	Mt Windsor	125	0.113
edible <30	Lauraceae	*E. grandis*	Ravenshoe S	Julatten	121	0.090
edible <30	Lauraceae	*E. grandis*	Bartle Frere	Mt Lewis	112	0.068
edible <30	Lauraceae	*E. grandis*	Bartle Frere	Julatten	106	0.087
edible <30	Lauraceae	*E. grandis*	Mt Baldy	Mt Lewis	84	0.099
edible <30	Lauraceae	*E. grandis*	Mt Baldy	Julatten	79	0.116
edible <30	Lauraceae	*E. grandis*	Eungella	Hidden Valley	64	0.153
edible <30	Lauraceae	*E. grandis*	Clarke Range	Hidden Valley	59	0.168
edible <30	Lauraceae	*E. grandis*	Julatten	Daintree	58	0.058
edible <30	Lauraceae	*E. grandis*	Mt Windsor	Daintree	56	0.057
edible <30	Lauraceae	*E. grandis*	Mt Lewis	Daintree	56	0.040
edible <30	Lauraceae	*E. grandis*	Julatten	Mt Windsor	54	0.071
edible <30	Lauraceae	*E. grandis*	Mt Lewis	Mt Windsor	47	0.049
edible <30	Lauraceae	*E. grandis*	Bartle Frere	Mt Baldy	44	0.064
edible <30	Lauraceae	*E. grandis*	Ravenshoe S	Bartle Frere	43	0.033
edible <30	Lauraceae	*E. grandis*	Ravenshoe S	Mt Baldy	42	0.059

edible <30	Lauraceae	*E. grandis*	Clarke Range	Eungella	9	0.025
edible <30	Lauraceae	*E. grandis*	Julatten	Mt Lewis	7	0.041
edible >30	Lauraceae	*Endiandra insignis*	Bolinda	Curtain Fig	77	0.255
edible >30	Lauraceae	*E. insignis*	Wooroonooran S	Curtain Fig	49	0.198
edible >30	Lauraceae	*E. insignis*	Bolinda	Wooroonooran S	29	0.147
edible >30	Lauraceae	*E. introrsa*	Dorrigo	Nightcap	210	0.242
edible >30	Lauraceae	*E. pubens*	Brunswick Heads	Bulburin	496	0.421
edible >30	Lauraceae	*E. pubens*	Nightcap	Bulburin	483	0.402
edible >30	Lauraceae	*E. pubens*	Hogan's Scrub	Bulburin	448	0.426
edible >30	Lauraceae	*E. pubens*	Brunswick Heads	Hogan's Scrub	49	0.108
edible >30	Lauraceae	*E. pubens*	Nightcap	Hogan's Scrub	44	0.076
edible >30	Lauraceae	*E. pubens*	Brunswick Heads	Nightcap	25	0.071
edible <30	Lauraceae	*Neolitsea dealbata*	Mt Warning	Junuy Juluum	236	0.224
edible <30	Lauraceae	*N. dealbata*	Border Ranges	Junuy Juluum	231	0.283
edible <30	Lauraceae	*N. dealbata*	Mt Warning	Glennifer	226	0.210
edible <30	Lauraceae	*N. dealbata*	Border Ranges	Glennifer	224	0.278
edible <30	Lauraceae	*N. dealbata*	Border Ranges 1	Junuy Juluum	220	0.247
edible <30	Lauraceae	*N. dealbata*	Nightcap	Junuy Juluum	220	0.212
edible <30	Lauraceae	*N. dealbata*	Broken Head	Junuy Juluum	215	0.265
edible <30	Lauraceae	*N. dealbata*	Border Ranges 1	Glennifer	212	0.236
edible <30	Lauraceae	*N. dealbata*	Nightcap	Glennifer	210	0.208
edible <30	Lauraceae	*N. dealbata*	Mt Warning	Bruxner Park	207	0.218
edible <30	Lauraceae	*N. dealbata*	Border Ranges	Bruxner Park	207	0.273
edible <30	Lauraceae	*N. dealbata*	Killen Falls	Junuy Juluum	205	0.251
edible <30	Lauraceae	*N. dealbata*	Broken Head	Glennifer	203	0.242
edible <30	Lauraceae	*N. dealbata*	Mt Warning	Nymboi-binderay	202	0.249
edible <30	Lauraceae	*N. dealbata*	Border Ranges	Nymboi-binderay	197	0.316
edible <30	Lauraceae	*N. dealbata*	Border Ranges 1	Bruxner Park	194	0.246
edible <30	Lauraceae	*N. dealbata*	Killen Falls	Glennifer	193	0.234
edible <30	Lauraceae	*N. dealbata*	Nightcap	Bruxner Park	189	0.210
edible <30	Lauraceae	*N. dealbata*	Nightcap	Nymboi-binderay	186	0.246
edible <30	Lauraceae	*N. dealbata*	Border Ranges 1	Nymboi-binderay	186	0.280
edible <30	Lauraceae	*N. dealbata*	Broken Head	Nymboi-binderay	182	0.288
edible <30	Lauraceae	*N. dealbata*	Broken Head	Bruxner Park	179	0.252
edible <30	Lauraceae	*N. dealbata*	Killen Falls	Nymboi-binderay	172	0.282
edible <30	Lauraceae	*N. dealbata*	Killen Falls	Bruxner Park	170	0.248

edible <30	Lauraceae	*N. dealbata*	Border Ranges	Broken Head	64	0.206
edible <30	Lauraceae	*N. dealbata*	Border Ranges	Killen Falls	62	0.188
edible <30	Lauraceae	*N. dealbata*	Border Ranges 1	Broken Head	53	0.165
edible <30	Lauraceae	*N. dealbata*	Border Ranges 1	Killen Falls	49	0.147
edible <30	Lauraceae	*N. dealbata*	Bruxner Park	Junuy Juluum	49	0.060
edible <30	Lauraceae	*N. dealbata*	Mt Warning	Killen Falls	48	0.113
edible <30	Lauraceae	*N. dealbata*	Mt Warning	Broken Head	47	0.130
edible <30	Lauraceae	*N. dealbata*	Nymboi-binderay	Bruxner Park	40	0.098
edible <30	Lauraceae	*N. dealbata*	Border Ranges	Nightcap	34	0.094
edible <30	Lauraceae	*N. dealbata*	Nymboi-binderay	Junuy Juluum	34	0.060
edible <30	Lauraceae	*N. dealbata*	Bruxner Park	Glennifer	32	0.043
edible <30	Lauraceae	*N. dealbata*	Nymboi-binderay	Glennifer	30	0.066
edible <30	Lauraceae	*N. dealbata*	Nightcap	Broken Head	30	0.111
edible <30	Lauraceae	*N. dealbata*	Nightcap	Killen Falls	28	0.081
edible <30	Lauraceae	*N. dealbata*	Border Ranges 1	Nightcap	24	0.075
edible <30	Lauraceae	*N. dealbata*	Mt Warning	Border Ranges	22	0.076
edible <30	Lauraceae	*N. dealbata*	Mt Warning	Border Ranges 1	20	0.062
edible <30	Lauraceae	*N. dealbata*	Mt Warning	Nightcap	20	0.051
edible <30	Lauraceae	*N. dealbata*	Glennifer	Junuy Juluum	17	0.035
edible <30	Lauraceae	*N. dealbata*	Border Ranges	Border Ranges 1	14	0.038
edible <30	Lauraceae	*N. dealbata*	Broken Head	Killen Falls	11	0.081
wind <30	Myrtaceae	*Tristaniopsis collina*	Nightcap	Darkwood	217	0.189
wind <30	Myrtaceae	*T. collina*	Border Ranges	Dorrigo	211	0.158
wind <30	Myrtaceae	*T. collina*	Border Ranges	Moonpar	200	0.171
wind <30	Myrtaceae	*T. collina*	Border Ranges	Orara West	200	0.198
wind <30	Myrtaceae	*T. collina*	Nightcap	Dorrigo	199	0.141
wind <30	Myrtaceae	*T. collina*	Nightcap	Moonpar	188	0.147
wind <30	Myrtaceae	*T. collina*	Nightcap	Orara West	185	0.180
wind <30	Myrtaceae	*T. collina*	Orara West	Darkwood	39	0.140
wind <30	Myrtaceae	*T. collina*	Moonpar	Darkwood	29	0.105
wind <30	Myrtaceae	*T. collina*	Border Ranges	Nightcap	27	0.160
wind <30	Myrtaceae	*T. collina*	Moonpar	Orara West	26	0.096
wind <30	Myrtaceae	*T. collina*	Orara West	Dorrigo	21	0.085
wind <30	Myrtaceae	*T. collina*	Dorrigo	Darkwood	19	0.101
wind <30	Myrtaceae	*T. collina*	Moonpar	Dorrigo	15	0.051
wind <30	Myrtaceae	*T. collina*	Border Ranges	Darkwood	228	0.210
wind <30	Myrtaceae	*T. laurina*	Tweed	Bellinger	231	0.134

wind <30	Myrtaceae	*T. laurina*	Border Ranges	Bellinger	228	0.129
wind <30	Myrtaceae	*T. laurina*	Border Ranges	Bonville	226	0.185
wind <30	Myrtaceae	*T. laurina*	Tweed	Bonville	225	0.186
wind <30	Myrtaceae	*T. laurina*	Tweed	Glennifer	221	0.130
wind <30	Myrtaceae	*T. laurina*	Border Ranges	Glennifer	221	0.123
wind <30	Myrtaceae	*T. laurina*	BarkersVale	Bellinger	218	0.107
wind <30	Myrtaceae	*T. laurina*	BarkersVale	Bonville	214	0.160
wind <30	Myrtaceae	*T. laurina*	NightcapLRC	Bellinger	210	0.106
wind <30	Myrtaceae	*T. laurina*	BarkersVale	Glennifer	209	0.106
wind <30	Myrtaceae	*T. laurina*	Tweed	Bobo	204	0.133
wind <30	Myrtaceae	*T. laurina*	KillenFalls	Bellinger	203	0.137
wind <30	Myrtaceae	*T. laurina*	Border Ranges	Bobo	203	0.121
wind <30	Myrtaceae	*T. laurina*	NightcapLRC	Bonville	203	0.156
wind <30	Myrtaceae	*T. laurina*	NightcapLRC	Glennifer	199	0.100
wind <30	Myrtaceae	*T. laurina*	Border Ranges	Bucca	199	0.142
wind <30	Myrtaceae	*T. laurina*	Tweed	Nymboida	198	0.143
wind <30	Myrtaceae	*T. laurina*	Tweed	Bucca	196	0.146
wind <30	Myrtaceae	*T. laurina*	Border Ranges	Nymboida	194	0.125
wind <30	Myrtaceae	*T. laurina*	KillenFalls	Bonville	192	0.190
wind <30	Myrtaceae	*T. laurina*	BarkersVale	Bobo	192	0.107
wind <30	Myrtaceae	*T. laurina*	KillenFalls	Glennifer	190	0.135
wind <30	Myrtaceae	*T. laurina*	BarkersVale	Bucca	186	0.121
wind <30	Myrtaceae	*T. laurina*	BarkersVale	Nymboida	184	0.108
wind <30	Myrtaceae	*T. laurina*	NightcapLRC	Bobo	183	0.105
wind <30	Myrtaceae	*T. laurina*	NightcapLRC	Nymboida	177	0.111
wind <30	Myrtaceae	*T. laurina*	KillenFalls	Bobo	175	0.136
wind <30	Myrtaceae	*T. laurina*	NightcapLRC	Bucca	174	0.120
wind <30	Myrtaceae	*T. laurina*	KillenFalls	Nymboida	171	0.144
wind <30	Myrtaceae	*T. laurina*	KillenFalls	Bucca	163	0.147
wind <30	Myrtaceae	*T. laurina*	Tweed	Cangai	145	0.143
wind <30	Myrtaceae	*T. laurina*	Border Ranges	Cangai	132	0.129
wind <30	Myrtaceae	*T. laurina*	KillenFalls	Cangai	131	0.143
wind <30	Myrtaceae	*T. laurina*	NightcapLRC	Cangai	128	0.113
wind <30	Myrtaceae	*T. laurina*	BarkersVale	Cangai	126	0.108
wind <30	Myrtaceae	*T. laurina*	Cangai	Bonville	119	0.151
wind <30	Myrtaceae	*T. laurina*	Cangai	Glennifer	109	0.093
wind <30	Myrtaceae	*T. laurina*	Cangai	Bellinger	108	0.089

wind <30	Myrtaceae	*T. laurina*	Cangai	Bucca	101	0.110
wind <30	Myrtaceae	*T. laurina*	Cangai	Bobo	90	0.082
wind <30	Myrtaceae	*T. laurina*	Cangai	Nymboida	76	0.079
wind <30	Myrtaceae	*T. laurina*	Border Ranges	KillenFalls	63	0.128
wind <30	Myrtaceae	*T. laurina*	Bucca	Bellinger	50	0.093
wind <30	Myrtaceae	*T. laurina*	BarkersVale	KillenFalls	48	0.104
wind <30	Myrtaceae	*T. laurina*	Nymboida	Bonville	45	0.146
wind <30	Myrtaceae	*T. laurina*	Tweed	KillenFalls	41	0.127
wind <30	Myrtaceae	*T. laurina*	Border Ranges	NightcapLRC	40	0.098
wind <30	Myrtaceae	*T. laurina*	Nymboida	Bucca	39	0.105
wind <30	Myrtaceae	*T. laurina*	Nymboida	Bellinger	34	0.087
wind <30	Myrtaceae	*T. laurina*	Nymboida	Glennifer	33	0.086
wind <30	Myrtaceae	*T. laurina*	Border Ranges	Tweed	31	0.118
wind <30	Myrtaceae	*T. laurina*	Bucca	Glennifer	30	0.082
wind <30	Myrtaceae	*T. laurina*	Bobo	Bonville	29	0.137
wind <30	Myrtaceae	*T. laurina*	Bucca	Bonville	29	0.140
wind <30	Myrtaceae	*T. laurina*	Bobo	Bellinger	28	0.074
wind <30	Myrtaceae	*T. laurina*	Bucca	Bobo	27	0.093
wind <30	Myrtaceae	*T. laurina*	BarkersVale	NightcapLRC	25	0.072
wind <30	Myrtaceae	*T. laurina*	Tweed	BarkersVale	24	0.091
wind <30	Myrtaceae	*T. laurina*	NightcapLRC	KillenFalls	23	0.101
wind <30	Myrtaceae	*T. laurina*	Tweed	NightcapLRC	22	0.091
wind <30	Myrtaceae	*T. laurina*	Bobo	Glennifer	19	0.071
wind <30	Myrtaceae	*T. laurina*	Nymboida	Bobo	16	0.059
wind <30	Myrtaceae	*T. laurina*	Border Ranges	BarkersVale	15	0.092
edible <30	Sapindaceae	*Doryphora australis*	Mt Warning	Orara	206	0.203
edible <30	Sapindaceae	*D. australis*	Mt Warning	Clouds SF	205	0.211
edible <30	Sapindaceae	*D. australis*	Brunswick Heads	Clouds SF	202	0.220
edible <30	Sapindaceae	*D. australis*	Border Ranges	Clouds SF	200	0.201
edible <30	Sapindaceae	*D. australis*	Border Ranges 1	Orara	200	0.205
edible <30	Sapindaceae	*D. australis*	Border Ranges 1	Clouds SF	196	0.214
edible <30	Sapindaceae	*D. australis*	Brunswick Heads	Orara	196	0.207
edible <30	Sapindaceae	*D. australis*	Hayters Hill	Clouds SF	190	0.269
edible <30	Sapindaceae	*D. australis*	Nightcap N	Clouds SF	189	0.217
edible <30	Sapindaceae	*D. australis*	Nightcap N	Orara	188	0.205
edible <30	Sapindaceae	*D. australis*	Nightcap S	Clouds SF	183	0.221
edible <30	Sapindaceae	*D. australis*	Hayters Hill	Orara	182	0.251

edible <30	Sapindaceae	*D. australis*	Nightcap S	Orara	180	0.209
edible <30	Sapindaceae	*D. australis*	Brunswick Heads	Washpool	141	0.131
edible <30	Sapindaceae	*D. australis*	Hayters Hill	Washpool	136	0.181
edible <30	Sapindaceae	*D. australis*	Mt Warning	Washpool	132	0.114
edible <30	Sapindaceae	*D. australis*	Washpool	Orara	126	0.227
edible <30	Sapindaceae	*D. australis*	Nightcap N	Washpool	123	0.114
edible <30	Sapindaceae	*D. australis*	Border Ranges	Washpool	120	0.091
edible <30	Sapindaceae	*D. australis*	Border Ranges 1	Washpool	120	0.101
edible <30	Sapindaceae	*D. australis*	Nightcap S	Washpool	118	0.138
edible <30	Sapindaceae	*D. australis*	Washpool	Clouds SF	96	0.236
edible <30	Sapindaceae	*D. australis*	Border Ranges	Hayters Hill	60	0.100
edible <30	Sapindaceae	*D. australis*	Clouds SF	Orara	50	0.068
edible <30	Sapindaceae	*D. australis*	Border Ranges	Brunswick Heads	50	0.043
edible <30	Sapindaceae	*D. australis*	Border Ranges 1	Hayters Hill	49	0.113
edible <30	Sapindaceae	*D. australis*	Mt Warning	Hayters Hill	43	0.122
edible <30	Sapindaceae	*D. australis*	Border Ranges 1	Brunswick Heads	40	0.060
edible <30	Sapindaceae	*D. australis*	Border Ranges	Nightcap S	39	0.058
edible <30	Sapindaceae	*D. australis*	Border Ranges	Nightcap N	34	0.017
edible <30	Sapindaceae	*D. australis*	Mt Warning	Brunswick Heads	30	0.058
edible <30	Sapindaceae	*D. australis*	Border Ranges 1	Nightcap S	28	0.064
edible <30	Sapindaceae	*D. australis*	Mt Warning	Nightcap S	27	0.066
edible <30	Sapindaceae	*D. australis*	Nightcap N	Hayters Hill	26	0.118
edible <30	Sapindaceae	*D. australis*	Nightcap S	Hayters Hill	25	0.103
edible <30	Sapindaceae	*D. australis*	Brunswick Heads	Nightcap S	24	0.054
edible <30	Sapindaceae	*D. australis*	Border Ranges 1	Nightcap N	23	0.021
edible <30	Sapindaceae	*D. australis*	Border Ranges	Mt Warning	21	0.032
edible <30	Sapindaceae	*D. australis*	Brunswick Heads	Nightcap N	21	0.056
edible <30	Sapindaceae	*D. australis*	Mt Warning	Nightcap N	20	0.039
edible <30	Sapindaceae	*D. australis*	Brunswick Heads	Hayters Hill	16	0.087
edible <30	Sapindaceae	*D. australis*	Border Ranges 1	Mt Warning	14	0.042
edible <30	Sapindaceae	*D. australis*	Border Ranges	Border Ranges 1	11	0.024
edible <30	Sapindaceae	*D. australis*	Nightcap N	Nightcap S	7	0.064
edible <30	Sapindaceae	*D. australis*	Border Ranges	Orara	206	0.191
edible >30	Sapotaceae	*Niemeyera prunifera*	Clarke Range	Robinson Creek	514	0.554
edible >30	Sapotaceae	*N. prunifera*	Clarke Range	Gooligans Creek	495	0.578
edible >30	Sapotaceae	*N. prunifera*	Clarke Range	Crawfords Lookout	492	0.595

edible >30	Sapotaceae	*N. prunifera*	Crawfords Lookout	Cape Tribulation	174	0.281
edible >30	Sapotaceae	*N. prunifera*	Gooligans Creek	Cape Tribulation	173	0.279
edible >30	Sapotaceae	*N. prunifera*	Robinson Creek	Cape Tribulation	168	0.239
edible >30	Sapotaceae	*N. prunifera*	Crawfords Lookout	Robinson Creek	33	0.146
edible >30	Sapotaceae	*N. prunifera*	Gooligans Creek	Robinson Creek	30	0.148
edible >30	Sapotaceae	*N. prunifera*	Crawfords Lookout	Gooligans Creek	4	0.099
edible >30	Sapotaceae	*N. prunifera*	Clarke Range	Cape Tribulation	655	0.584
edible >30	Sapotaceae	*N. whitei*	Brunswick Heads	Ulidarra	196	0.309
edible >30	Sapotaceae	*N. whitei*	Nightcap	Ulidarra	180	0.331
edible >30	Sapotaceae	*N. whitei*	Nightcap	Brunswick Heads	23	0.302
edible >30, small seed	Sapotaceae	*Planchonella australis*	Dorrigo	Nightcap	202	0.068
edible >30, small seed	Sapotaceae	*P. australis*	Dorrigo	Mary	401	0.092
edible >30, small seed	Sapotaceae	*P. australis*	Hayters Hill	Nightcap	25	0.067
edible >30, small seed	Sapotaceae	*P. australis*	Hayters Hill	Mary Cairncross	222	0.097
edible >30, small seed	Sapotaceae	*P. australis*	Nightcap	Mary Cairncross	212	0.076
edible >30, small seed	Sapotaceae	*P. australis*	Dorrigo	Hayters	207	0.092
edible <30	Sapotaceae	*Pleioluma queenslandica*	Brunswick Heads	Eungella	967	0.220
edible <30	Sapotaceae	*P. queenslandica*	Cooloolah	Eungella	711	0.166
edible <30	Sapotaceae	*P. queenslandica*	Brunswick Heads	Byfield	697	0.146
edible <30	Sapotaceae	*P. queenslandica*	Bulburin	Eungella	497	0.113
edible <30	Sapotaceae	*P. queenslandica*	Brunswick Heads	Bulburin	479	0.115
edible <30	Sapotaceae	*P. queenslandica*	Cooloolah	Byfield	424	0.091
edible <30	Sapotaceae	*P. queenslandica*	Byfield	Eungella	297	0.098
edible <30	Sapotaceae	*P. queenslandica*	Brunswick Heads	Cooloolah	291	0.135
edible <30	Sapotaceae	*P. queenslandica*	Bulburin	Byfield	218	0.056
edible <30	Sapotaceae	*P. queenslandica*	Cooloolah	Bulburin	216	0.069
