# Supplementary figures and images for: Genomic Screening to Identify Food Trees Potentially Dispersed by Precolonial Indigenous Peoples
Source: Genes (Basel). 2022 Mar 8;13(3):476. doi: 10.3390/genes13030476 (PMC8954434; doi:10.3390/genes13030476)

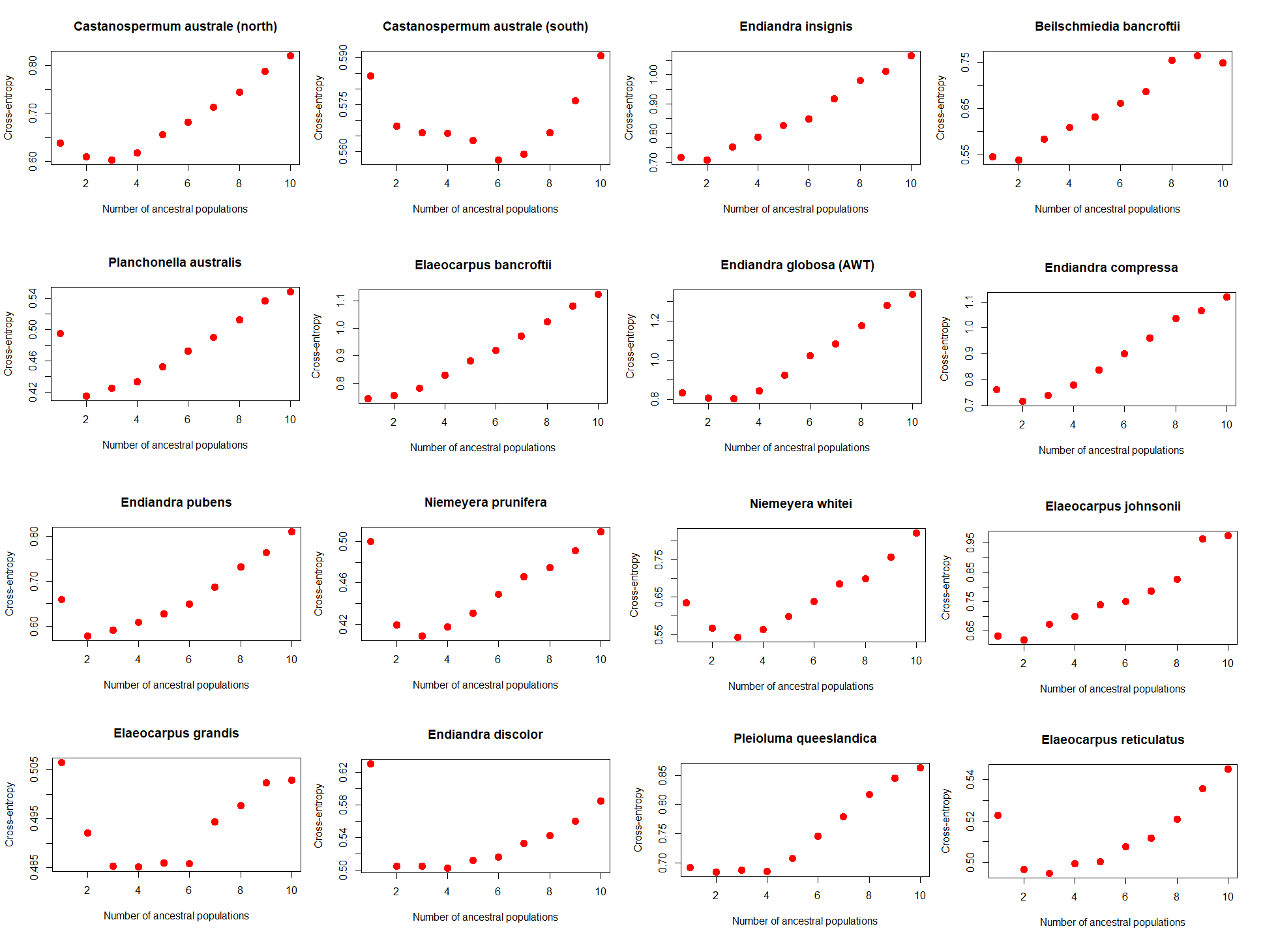

Supplement: Supplementary file 1 [file genes-13-00476-s001.zip › Figure_S1.tif]
